# Bioactivities evaluation of an endophytic bacterial strain *Bacillus velezensis* JRX-YG39 inhabiting wild grape

**DOI:** 10.1186/s12866-022-02584-0

**Published:** 2022-07-02

**Authors:** Baozhen Feng, Dandan Chen, Ruixue Jin, Erqin Li, Peiqian Li

**Affiliations:** Key Laboratory of Plant Disease and Pest Control, Department of Life Science, Yuncheng University, Yuncheng, 044000 People’s Republic of China

**Keywords:** Endophyte, *Botrytis cinerea*, Wild grape, Antifungal activity, Growth promotion, VOCs

## Abstract

**Background:**

*Botrytis cinerea* can cause serious disease on lots of plant hosts during growth and postharvest storage. Biocontrol is known to be eco-friendly methods to control pathogens. Plant endophytic bacteria are generally considered as beneficial organisms, since they can promote plant growth and enhance plant immune system. Thus, screening biological control agents is very important for sustainable plant protection.

**Results:**

Fifty-six endophytic bacteria were obtained from wild grape. Sixteen isolates and their extracts exhibited significant antifungal activity against *B. cinerea*. Particularly, strain JRX-YG39 with the strongest inhibition ability had a broad-spectrum antifungal activity. Combining 16S rDNA analysis and the phylogenetic results based on *gyr*A and *gyr*B genes, JRX-YG39 was assigned as *Bacillus velezensis*. JRX-YG39 could produce bioactive VOCs and obviously depressed mycelia growth of *B. cinerea*. It was confirmed that VOCs released by JRX-YG39 could significantly promote growth and induce defense of *Arabidopsis thaliana*. Thirty-one bioactive secondary metabolites were further identified from JRX-YG39 culture by gas chromatography–mass spectrometry analysis. Dibutyl phthalate, a potential antifungal substance, was the major compound accounting for 78.65%.

**Conclusions:**

*B. velezensis* JRX-YG39 has wide broad-spectrum antagonistic activity and significant plant promotion activity. Hence, *B. velezensis* JRX-YG39 will provide a valuable constituent of modern agricultural practice as biofertilizers and biocontrol agents.

**Supplementary Information:**

The online version contains supplementary material available at 10.1186/s12866-022-02584-0.

## Background

*Botrytis cinerea* can cause serious disease on more than 1400 plant hosts during growth and postharvest storage [1]. Chemical control is still a major measure to prevent grey mold on plant. However, *B. cinerea* has developed multiple fungicide resistance due to its high genetic variability and rapid reproduction [2, 3]. Additionally, the spraying fungicides in large quantities poses a great threat to the environment and human safety.

Biocontrol including the use of biological control agents and their active compounds has been considered as a sustainable approach to control plant diseases [4–6]. Plant endophytic bacteria are generally considered as beneficial organisms, since they can promote plant growth and enhance plant immune system [7, 8]. In recent years, lots of reports indicated that endophytic bacteria strains from genus *Bacillus* displayed significant potential in biological control of gray mold. The *Bacillus* genus possess genetic biodiversity and has been considered as biocontrol candidates against pathogens. For example, *B. subtilis* strains MBI 600 and QST 713 has been registered for the use against plant diseases, especially the latter was particularly effective against *B. cinerea* [9]. Meanwhile, *B. amyloliquefaciens* could prevent fruit diseases and plant pathogens such as *B. cinerea*, *Monilinia fructicola*, *M. laxa*, *Penicillium digitatum*, *P. expansum* and *P. italicum* [9–11]. *B. halotolerans* successfully inhibited *B. cinerea*, *Fusarium oxysporum* f. sp. radicis-lycopersici, *Alternaria alternata*, *Rhizoctonia bataticola*, and *Phytophthora infestans* [12, 13]. Recently, several studies have confirmed that *B. velezensis* is a valuable member of significant bioactivity on both pathogen suppression and plant growth promotion [14, 15].

Accumulated evidence has demonstrated that endophytes can produce active secondary metabolites including soluble substances and volatile organic compounds (VOCs). *Bacillus* are capable of produce a spectrum of bioactive secondary metabolites including nonribosomal peptide synthetases and polyketide synthases [15–19]. Indeed, VOCs produced by bacteria in *Bacillus*, *Pseudomonas* and *Enterobacter* genera demonstrated significantly antifungal activity. These VOCs included terpenes, esters, ketones, hydrocarbons, sulfur derivatives, acids, etc. [20, 21]. Several studies indicated that the plant growth promoting ability of endophytic bacteria had host specificity [22, 23]. The objectives of our study are to 1) isolation and identification of endophytic bacteria with bioactivity inhabiting wild grape in Zhong-tiao mountains; 2) determine the antagonistic activity and plant promotion activity of strain JRX-YG39; 3) identified the predominant volatile antifungal compounds from JRX-YG39, and elucidated the inhibitory mechanism.

## Results

### Isolation and identification of endophytic bacteria from wild grape

In total, 56 endophytic bacteria were isolated from wild grape tissue using NA medium. All the isolates were performed preliminary screening for antifungal activity. By antagonistic assay in plates, 16 isolates showed significant activities, accounting for 28.57% of the total isolates (Table1). According to the 16 S rRNA alignment results, most strains belonged to *Bacillus velezensis*. As shown in Table 1 and Fig. S1, 16 isolates could good control mycelia growth of *B. cinerea*, among which seven isolates owned more than 50% of inhibition activity. Five isolates showed more than 50% of inhibition rate against *A. alternata*. Strains JRX-YG39 and YB-K1 could obviously suppress *F. pernambucanum* growth with inhibition rate more than 50%. In addition, strains JRX-YG39, JRX-QT40, and YB-K1 could control *C. gloeosporioides* with inhabitation rate were 81.91%, 51.23%, and 66.74%, respectively (Fig. S1). Particularly, strain JRX-YG39 demonstrated excellent antifungal activity and was selected for the subsequent study.

**Table 1:** Primary identification of endophytic bacteria inhabiting wild grapes

Strains	16 S rRNA Accession number	Identity to closest species (Acc.No.)	Antifungal activity			
			*B. cinerea*	*A. alternata*	*F*. *pernambucanum*	*C. gloeosporioides*
JRX-YJF1	ON413862	99.65% to *B. subtilis* (NR_027552.1)	+	+	+	-
JRX-YJF2	ON413863	99.51% to *B. subtilis* (NR_027552.1)	+	+	+	+
JRX-YJF3	ON413864	99.79% to *B. subtilis* (NR_027552.1)	+	+	+	-
JRX-YJF4	ON413865	99.79% to *B. tequilensis* (NR_104919.1)	+	+	+	+
JRX-YJF5	ON413866	99.65% to B. *tequilensis* (NR_104919.1)	+	+ +	+	-
JRX-YP5	ON413867	99.38% to *B. velezensis* (NR_075005.2)	+	+	+	+
JRX-YP8	ON413868	99.58% to *B. velezensis* (NR_075005.2)	+ +	+ +	+	+
JRX-YG28	ON413869	99.58% to *B. velezensis* (NR_116240.1)	+ +	+ +	+	+
JRX-YG29	ON413870	99.79% to *B. tequilensis* (NR_104919.1)	+ +	+	+	+
JRX-YG 39	ON413871	99.65% to *B. velezensis* (NR_116240.1)	+ + +	+ + +	+ + +	+ + +
JRX-QT36	ON413872	99.65% to *B. subtilis* (NR_113265.1)	+ +	+	+	+
JRX-QT40	ON413873	99.38% to *B. velezensis* (NR_116240.1)	+	+	+	+ +
JRX-YP13	ON413874	99.31% to *B. velezensis* (NR_116240.1)	+ +	+	+	+
JRX-YP14	ON413875	99.58% to *B. velezensis* (NR_116240.1)	+	+	+	+
JRX-YP15	ON413876	99.38% to *B. velezensis* (NR_075005.2)	+	+	+	-
YB-K1	MW642498	99.65% to *B. velezensis* (NR_116240.1)	+ +	+ +	+ +	+ +

Antifungal activity was tested by confrontation culture for endophytic bacteria and plant pathogens *Botrytis cinerea, Fusarium pernambucanum, Alternaria alternata,* and *Colletotrichum gloeosporioides*, respectively*.* “ + ” indicated inhibition rate below 50%, “ +  + ” indicated inhibition rate between 50%—80%, “ +  +  + ” indicated inhibition rate above 80%, and “-” indicated no antifungal activities

### Identification of strain JRX-YG39

Endophytic bacterium JRX-YG39 was identified using sequencing of 16S rRNA, DNA gyrase subunit A gene (*gyr*A), and DNA topoisomerase (ATP-hydrolyzing) subunit B gene (*gyr*B). The 16S rRNA, *gyr*A, and *gyr*B were deposited in Genbank with accession numbers ON413871, ON507281, and ON507282, respectively.

**Fig. 1:**
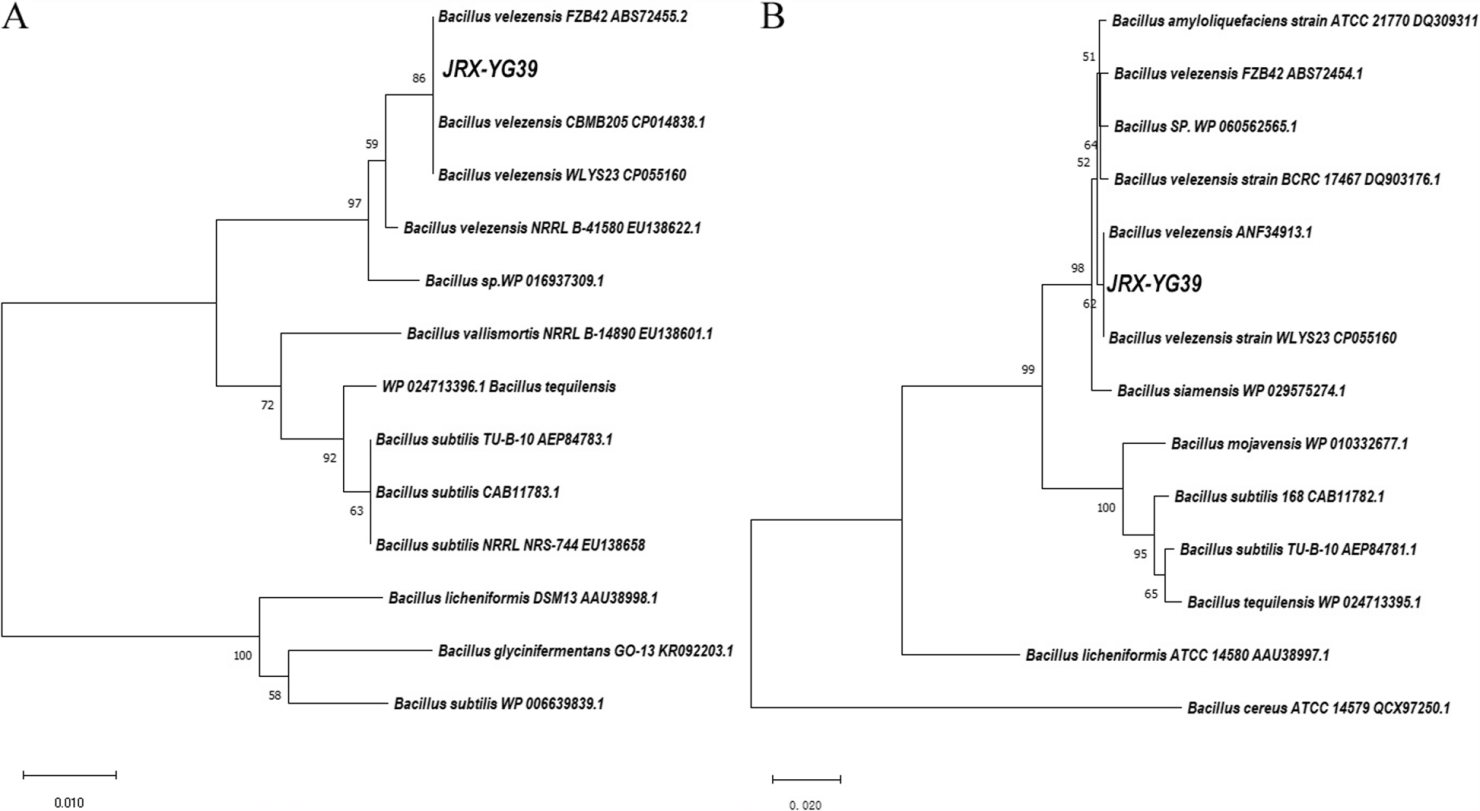
Phylogenetic trees of JRX-YG39 based on *gyr*A and *gyr*B genes. **A** Phylogenetic trees of JRX-YG39 based on *gyr*A; **B** Phylogenetic trees of JRX-YG39 based on *gyr*B

We performed 16S rRNA sequence alignment with type strains on NCBI. The result indicated that the strain JRX-YG39 demonstrated 99.65% identity to *B. velezensis* (NR_116240.1) (Table 1). To further determine the classification of JRX-YG39, we constructed neighbor-joining tree based on *gyr*A and *gyr*B genes. As shown in Fig. 1, both *gyr*A and *gyr*B phylogenetic trees demonstrated that JRX-YG39 was affiliated with *B. velezensis.* Thus, JRX-YG39 was identified as *B. velezensis* and named after *B. velezensis* JRX-YG39.

### Antifungal activity assay of JRX-YG39 fermentation extract

**Fig. 2:**
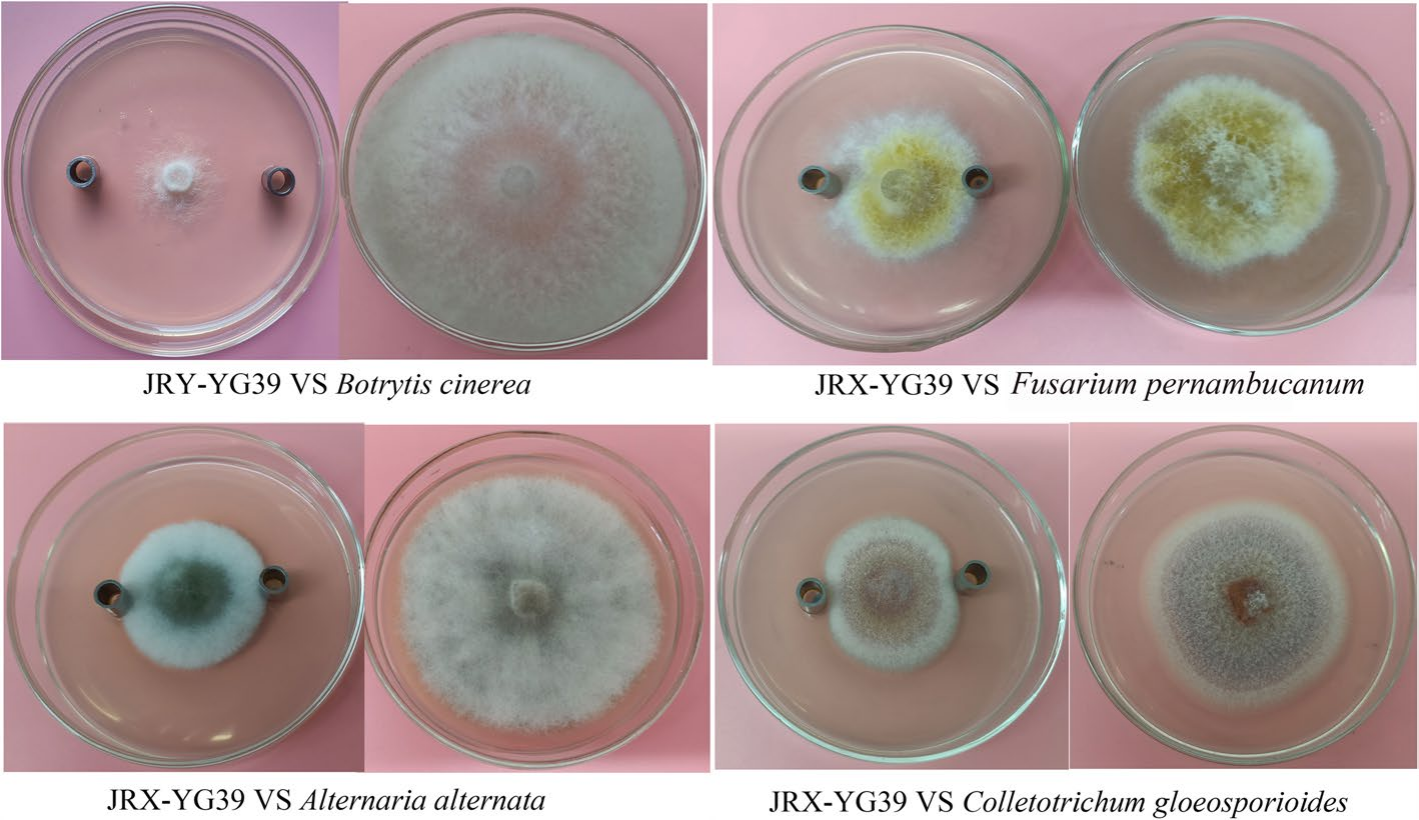
Antifungal activity of *B. velezensis* JRX-YG39 culture against four plant pathogens on plates

Oxford cup experiment results demonstrated that strain *B. velezensis* JRX-YG39 fermentation broth owned antifungal substances (Fig. 2). Compared with control group, pathogen colonies in treatment were obviously smaller and sparser, which indicated that the bacterial strain could significantly suppress mycelium growth. The inhibition rates ranged from 50 to 85%. The maximal inhibition rates were observed against *B. cinerea* (85.48% ± 1.87).

### Bioactivity of VOCs released by JRX-YG39

**Fig. 3:**
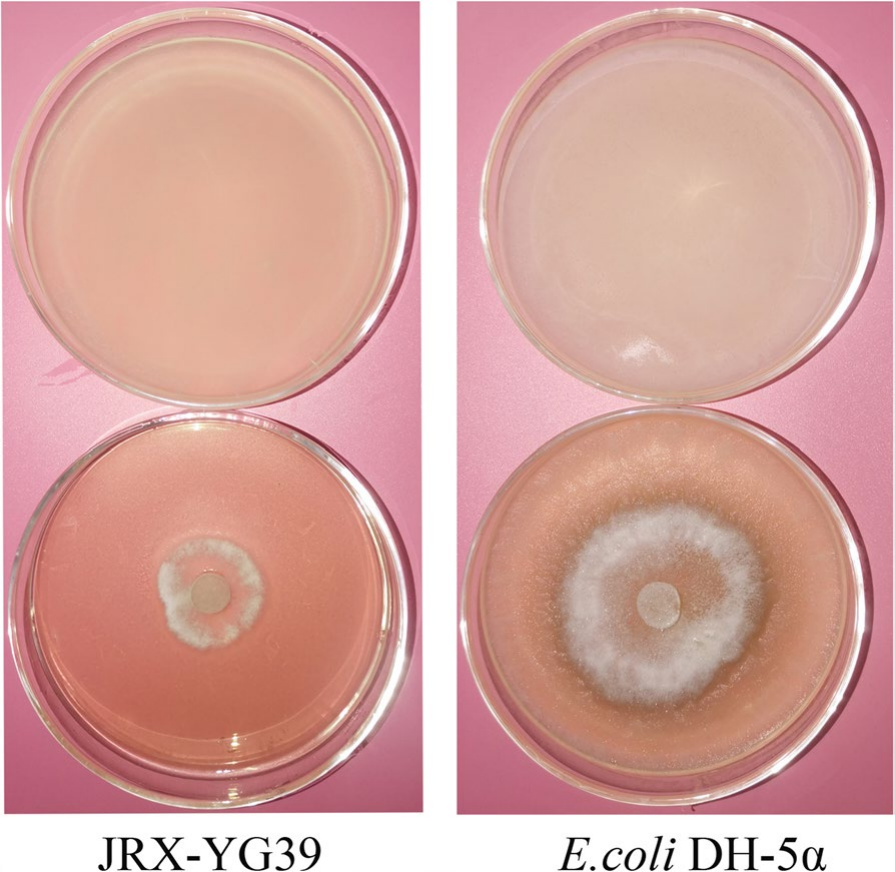
VOCs released by *B. velezensis* JRX-YG39 demonstrated strong inhibition against *B. cinerea*

Double plate assay indicated that strain JRX-YG39 could produce antagonistic activity VOCs inhibiting growth of *B. cinerea* (Fig. 3). Compared with *Escherichia coli* DH-5α, JRX-YG39 could influence *B. cinerea* growth with inhibition rate up to 80%. While *B. cinerea* had grown all over the plate in the control group.

### Growth promotion effect of JRX-YG39 VOCs on *A. thaliana* seedlings

**Fig. 4:**
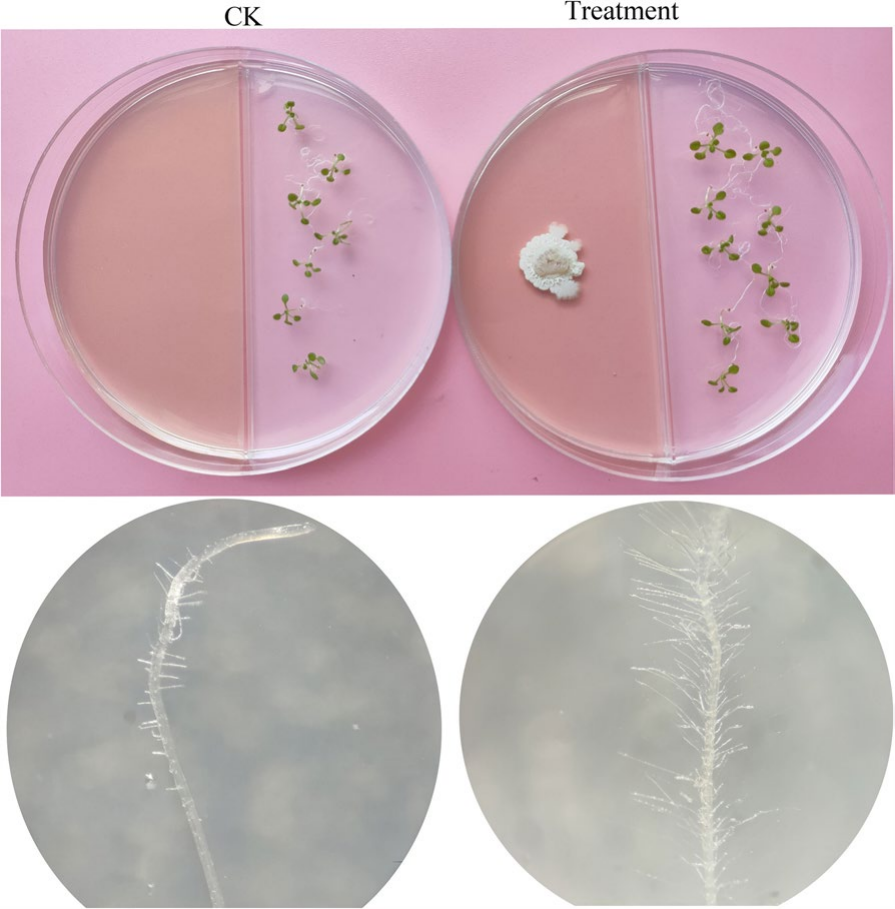
VOCs released by *B. velezensis* JRX-YG39 demonstrated significant growth promotion on leaves and roots of *A. thaliana* seedlings

**Fig. 5:**
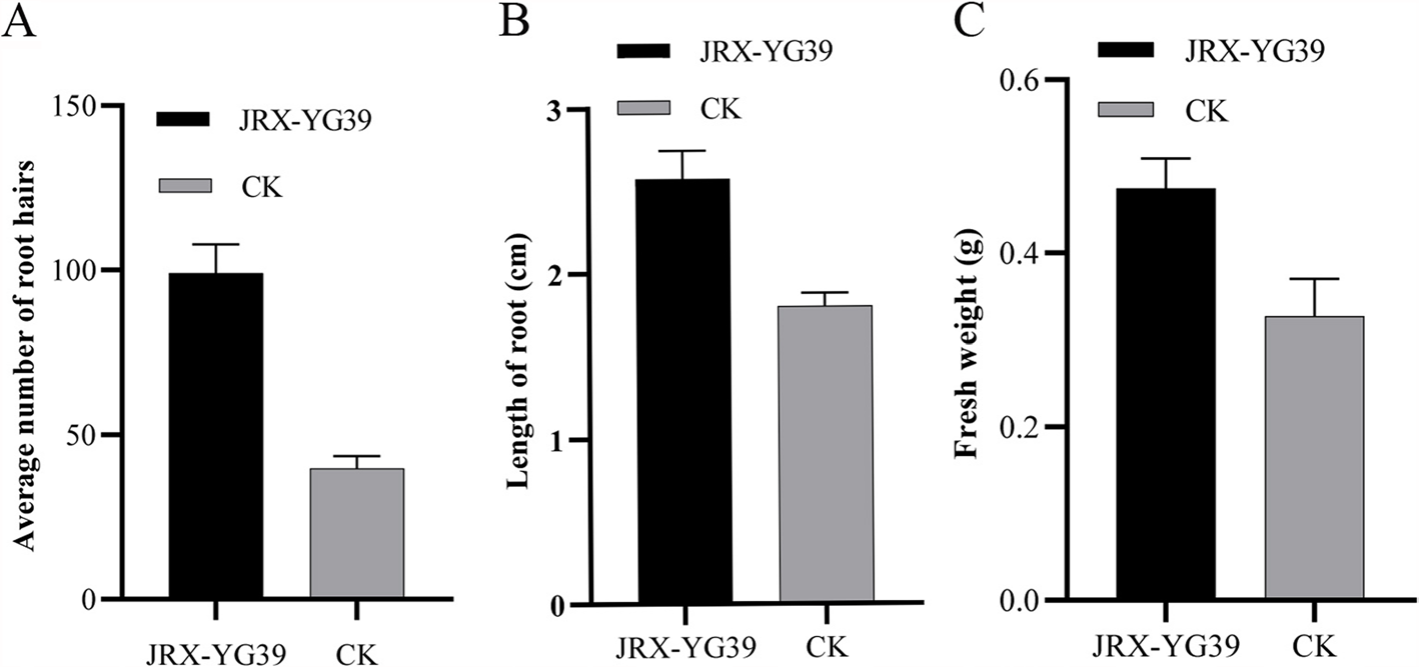
Analysis of data on growth index of *A. thaliana* seedlings treated with JRX-YG39. **A** Average number of root hairs of *A. thaliana* seedlings; **B** Root length of *A. thaliana* seedlings; **C** Fresh weight of *A. thaliana* seedlings

Plant growth promotion effect of *B. velezensis* JRX-YG39 was measured in Figs. 4 and 5. By contrast, *A. thaliana* seedlings demonstrated increased growth with dark green leaves, longer main root, and developed lateral roots. The endophytic strain significantly (LSD’s multiple range test, *p* < 0.05) enhanced number of lateral roots, main root length, and the fresh weight after culture for three weeks (Fig. 5). Compared to control (about 42), the average number of lateral roots treated with JRX-YG39 was 100 (Fig. 5A). The length of main root in treatment group was 2.6 cm, while the control group was only 1.8 cm (Fig. 5B). In addition, and the fresh weight of *A. thaliana* plantlets treated with *B. velezensis* JRX-YG39 reached 0.48 g, which was much greater than that in control (Fig. 5C).

### Resistance improvement effect of JRX-YG39

**Fig. 6:**
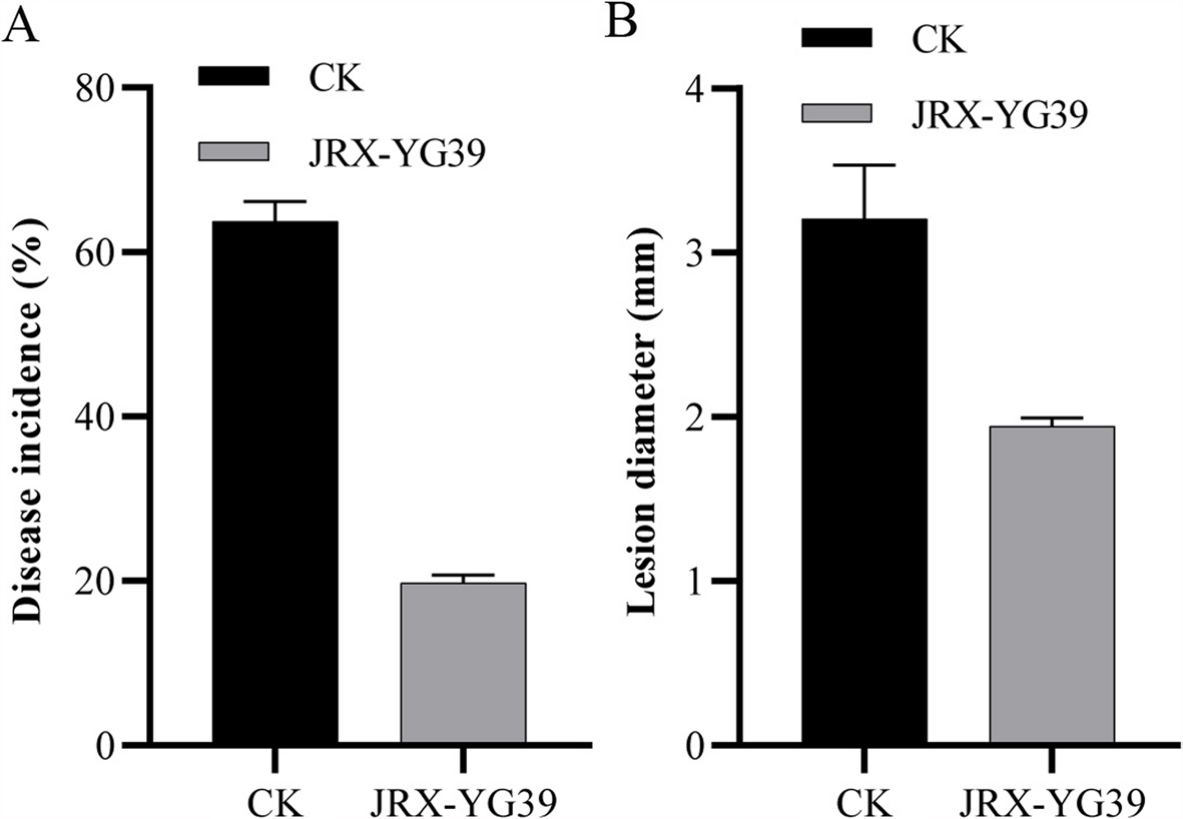
Disease incidence (**A**) and diameter of lesions (**B**) in *A. thaliana* after inoculation with *B. cinerea* conidia suspension

After incubation, *A. thaliana* plantlets were challenged with *B. cinerea* conidia suspension for resistance test. Compared with control group, *A. thaliana* plantlets treated with JRX-YG39 showed obviously lower disease incidence with 19.38% (Fig. 6 A; Fig. S2). Lesion diameter of treated group was 1.9 mm, and control group had bigger lesion about 3.2 mm in diameter (Fig. 6B). The results illustrated VOCs released by JRX-YG39 had induced plantlet resistance contributing to decrease of disease incidence and lesion diameter.

### Identification of VOCs of JRX-YG39 culture by gas chromatography–mass spectrometry

Fermentation extract of *B. velezensis* JRX-YG39 was conducted GC–MS assay under optimized conditions. Assay results showed that the VOCs contained 31 substances including alcohols, aldehydes, olefins, organic acids, and lipids, etc. (Table S1). According to NIST library data, main chemical compounds with area percent more than 1% were identified as 3-methyl-butanoic acid, 2-methyl-butanoic acid, 3,5-dimethoxy- phenol, dibutyl phthalate, phenol, 2-Nonanone, l-leucine-N-cyclopropylcarbonyl-hexadecyl-ester, [1,2-a]pyrazine-1,4-dione-hexahydro-3-(2-methylpropyl)-Pyrrolo, (S-E)-2,3,7-trimethyl-4-Octene, and (Z)-9-octadecenamide. Particularly, results revealed a high content of dibutyl phthalate (78.65%) among all compounds.

## Discussion

Although vast documentation on the pathogenic mechanisms and control have deposited, *B. cinerea* remains a remarkable challenge to overcome and promote its biocontrol. Therefore, isolation and evaluation microbe with broad-spectrum bioactive is essential for sustainable plant protection. Endophytes are considered as important high potential biological control agents, and have been found widely in various plants [24]. Several reports have indicated that plant living different environment could own diverse not only the soil and rhizosphere [25] but also phyllosphere [26] and endosphere communities [27]. Exploration of wild plant endophytes will provide new candidates for development of new and effective bioactive compounds in further plant disease management.

In this study, we isolated 16 endophytic bacteria with antifungal activity against four pathogens from wild grape in Zhong-tiao mountain. Most of these isolates belonged to *Bacillus* spp. based on 16S rRNA. Strain JRX-YG39 was identified as *B. velezensis* according to 16S rRNA, *gyrA* gene, and *gyrB* gene sequence analysis. JRX-YG39 demonstrated strong antifungal activity against *B. cinerea* by producing soluble and diffusible substances. Endophytic *Bacillus* spp. with antimicrobial activity have been documented in other studies. *B. amyloliquefaciens* possessed obvious inhibition effect on *Fusarium oxysporum*, *Penicillium* spp*.*, *Colletotrichum* spp., *B. cinerea*, and *Sclerotinia sclerotiorum* [28, 29]*. B. halotolerans* could suppress mycelia growth of *B. cinerea* and *F. oxysporum* [30, 31]. In addition, *B. velezensis* is a valuable species as potential biocontrol agent against many phytopathogens. For example, endophytic *B. velezensis* B-36 isolated from lotus tissues showed strong antagonistic activities against lotus rot caused by *F. oxysporum* [14]. *B. velezensis* strain SDTB038 displayed good control of potato late blight (*Phytophthora infestans*) in greenhouses and field [32]. Strain JRX-YG39 could greatly inhibit the growth of four important phytopathogens, particularly inhibitory rate reached to 87.41% when treatment against *B. cinerea* on plate*. Bacillus* species can produce a set of bioactive secondary metabolites that potentially control plant pathogens [15].

,Strain JRX-YG39 significantly enhanced *A. thaliana* growth and triggered plant defense response by producing bioactive VOCs. Accumulated documents indicated that many bacteria displayed prominent plant growth promotion effect [15, 33]. Similar reports showed that endophytic *Bacillus* strains from tomato could obviously promote growth of tomato seedlings [34]. Additionally, growth promotion effects on *A. thaliana* roots were much stronger than that in leaves when incubation with strain JRX-YG39 (Fig. 4). In another study, *B. subtilis* strain GB03 displayed much stronger enhancement on roots elongation of *A. thaliana* seedlings compared to leaves [35]. Furthermore, it has been known that *Bacillus* can stimulate induced plant systemic resistance by producing various substances [8, 15, 36, 37]. In this study, disease incidence of *A. thaliana* seedlings challenged with *B. cinerea* decreased significantly, compared to control group. Lesion diameter of diseased leaves was about 1.9 mm, which was much smaller than that in control treatment. These results indicated JRX-YG39 could successfully boost plant defenses and decrease disease incidence.

In total, 31 bioactive secondary metabolites were further identified from the *B. velezensis* JRX-YG39 fermentation extract by GC–MS. Particularly, dibutyl phthalate was the major compound accounting for 78.65%. Dibutyl phthalate was reported as strongly antifungal compound in several actinomycete strains [38, 39]. Another study reported that dibutyl phthalate from marine *Pseudomonas* could inactivate cathepsin B [40]. Phenol and hexadecane were critical for antifungal activity to *B. cinerea* and *A. alternaria* [41]. VOCs released by *Bacillus* sp. BCT9 contained 2-Nonanone that leading to increase of lateral root length on *Lactuca sativa* seedlings [42]. Function of other bioactive substances emitted by JRX-YG39 need to be demonstrated in further study. Thus, *B. velezensis* JRX-YG39 could be selected as potential biocontrol agents to manage *B. cinerea*.

## Conclusion

Endophytic bacterium *B. velezensis* JRX-YG39 could produce active soluble and volatile compounds contributing to antifungal activity, plant growth promotion, and resistance enhance effect. It had a great potential to provide novel agents for gray mold management in future.

## Materials and methods

### Microbes and plants

Four pathogens *Botrytis cinerea* LK-7, *Fusarium pernambucanum* FQ-17, *Alternaria alternata* YJHB-6, and *Colletotrichum gloeosporioides* PG-10 were isolated from diseased plants and stored in refrigerator at 4℃. Wild grape (*Vitis heyneana* Roem. et Schult) samples were obtained from the primitive ecological area of Zhong-Tiao Mountains in Shanxi Province, China (latitude 35^◦^09′ N, longitude 111^◦^20′ E, and elevation 400 m). *Arabidopsis thaliana* seeds were gift from Chinese Academy of Agricultural Sciences.

### Isolation, screening and identification of endophytic bacteria from wild grape

Endophytic bacteria were isolated from wild grape samples by using nutrient agar (NA) medium according to the method of described previously [43]. The isolated strain was maintained in glycerol solution (30%) at − 20^◦^C.

The genomic DNA of candidate strains were extracted using a bacterial genomic DNA isolation kit (Biotech Corporation, Beijing, China). The harvested DNA was detected by the agarose gel electrophoresis and quantified by Qubit® 2.0 Fluorometer (Thermo Scientific). 16S rRNA was amplified using the universal primers 27 F and 1492R according to previous studies [41]. Candidate strains were preliminarily identified by alignment of 16S rRNA sequences with BLAST online software (https://blast.ncbi.nlm.nih.gov/Blast).

### Phylogenetic trees construction of JRX-YG39 based on *gyr*A and *gyr*B genes

To further distinguish strain JRX-YG39, DNA gyrase subunit A gene (*gyr*A) and DNA topoisomerase (ATP-hydrolyzing) subunit B gene (*gyr*B) were amplified according to previous researches [32, 41]. Amplification products were sequenced and deposited in NCBI. Then sequences alignment was performed with CLUSTAL W in Mega-X software [44]. Phylogenetic trees were constructed based on *gyr*A and *gyr*B according to the neighbour-joining approach with bootstrap values based on 1000 replications, respectively.

### Antifungal activity assay of JRX-YG39 fermentation extract

After preliminary screening of antifungal strains, JRX-YG39 was selected for further study. Strain JRX-YG39 was cultured with liquid NA medium in a 150-mL flask at 30 ℃, 180 rpm, for 24 h. The fermentation broth was collected and was centrifuged for 10 min at 4000 × g to collect supernatant. Then Oxford cup experiment was performed as follows: a 5-mm hyphal disk of plant pathogen (*Botrytis cinerea*, *Fusarium pernambucanum*, *Alternaria alternata*, and *Colletotrichum gloeosporioides*) was placed in the middle of a PDA plate, then two Oxford cups were placed in each side of about 2.5-cm distance from the pathogen, 100 µL of JRX-YG39 supernatant was added in the Oxford cups. Plates were incubated at 24 ^◦^C in dark for 5–7 days. The inhibition percentage was evaluated according to the method of Gao et al. [41]. Control Petri dishes contained only two mycelia disks of the fungal strains. The experiment was repeated three times.

### Double plate assay of VOCs released by JRX-YG39

Double plate assay was used to study antagonistic activity of volatile organic compounds (VOCs) against *B. cinerea* according to methods in previous study [41]. A 5-mm hyphal disk of *B. cinerea* culture was placed in the middle of a PDA plate. Then a NA plate spread with 20 µL of JRX-YG39 culture was inverted over the pathogen plate and incubated. Control treatment consisted of mycelia disc of pathogen and *Escherichia coli* DH-5α. Plates were sealed with cling wrap and incubated at 24 ^◦^C in dark 5–7 days. The inhibition percentage was calculated as described earlier.

### Co-culture *A. thaliana* seedlings with JRX-YG39

According to previous study [42], 10 mL of Murashige and Skoog medium (MS) and NA medium were poured into a two-compartment petri dish, respectively. After medium solidification, sterilized *A. thaliana* seeds were planted on the MS medium. Then a disk of strain JRX-YG39 was put at the middle of NA medium. The plates without bacteria were used as control. The seedlings were cultivated in a growth chamber with a 16/8-h day/night schedule at 20 ^◦^C. After 21 days, the fresh weight, length of root, number of root hairs were measured. Each treatment consisted of three replicates.

### Inoculation *A. thaliana* seedlings with *B. cinerea*

After treatment with bacterial strain JRX-YG39 as described above, *A. thaliana* seedlings were inoculated with *B. cinerea* conidia suspension (1 × 10^6^/ mL) by a 1-mL syringe. Then disease incidence and lesion diameter on leaves were calculated. Each treatment consisted of twenty seedlings.

### Analyses of VOCs by gas chromatography–mass spectrometry

The strain JRX-YG39 was streaked in NA medium at 30℃ for 10 h. Single colony was cultured in 100 mL LB liquid medium at 30℃, 200 RPM /min for 24 h. Then 20 µL JRX-YG39 suspension was transferred to 300 mL LB at 30℃, 200 RPM /min for 48 h. Next, the fermentation liquid was extracted with ethyl acetate (1:1) for 3 times, and the extraction liquid was concentrated by rotary evaporation at 35℃.

Subsequently, the strain extract was first dissolved in the chromatographic-grade methanol and filtered through a 0.2-µm filter. The solution was injected into a gas capillary column (DB-17MS, 30 m × 0.25 mm × 0.25 µm) of a gas chromatography (5975C Inert XL MSD, Agilent, United States). Helium was used as a carrier gas with a flow rate of 1 ml min^−1^. The mass spectrometer (EI with replaceable horn) was operated in the electron ionization (EI) mode at 70 eV with a continuous scan from 50 to 800 m/z. The peaks were identified by matching the mass spectra with the National Institute of Standards and Technology (NIST, United States) library.

### Statistical analysis

All data were analyzed using analysis of variance (ANOVA) by the SPSS statistical package (version 22, SPSS Inc., Chicago, IL, United States). The t-test was applied to compare means between different subjects. The statistical differences between means were assessed at the level of *p* < 0.05.

## Supplementary Information

**Additional file 1: Fig. S1.** Antifungal activity of 16 endophytic bacteria against four phytopathogens. A to D represented growth inhibition of Botrytis cinerea, Alternaria alternata, Fusarium pernambucanum, and Colletotrichum gloeosporioides after antagonist with 16 endophytic bacteria, respectively. **Fig. S2.** Symptom difference on A. thaliana seedlings treatment with B. cinerea conidia suspension. A. A. thaliana seedlings co-cultured with B. velezensis JRX-39 for 21 days and then inoculated with B. cinerea; B. control, A. thaliana seedlings cultured for 21 days on MS medium and inoculated with B. cinerea conidia suspension, showed more severe disease. Arrows indicated diseased leaves. **Table S1.** Identification of VOCs of the Bacillus JRX-YG39 by GC-MS.

## Data Availability

All data generated or analyzed during this study are included in this published article. and supplementary material online. The 16S rRNA sequence of endophytic bacteria generated and analysed during the current study are available in the GenBank repository with accession number ON413862—ON413876, and MW642498. *gyr*A, and *gyr*B sequences of JRX-YG39 strain were also deposited in GenBank with accession numbers ON507281 and ON507282, respectively.
