# Supplementary material for: Bioactivities evaluation of an endophytic bacterial strain Bacillus velezensis JRX-YG39 inhabiting wild grape
Source: BMC Microbiol. 2022 Jul 2;22:170. doi: 10.1186/s12866-022-02584-0 (PMC9250181; doi:10.1186/s12866-022-02584-0)
Supplement: Supplementary file 1 — Additional file 1: Fig. S1. Antifungal activity of 16 endophytic bacteria against four phytopathogens. A to D represented growth inhibition of Botrytis cinerea, Alternaria alternata, Fusarium pernambucanum, and Colletotrichum gloeosporioides after antagonist with 16 endophytic bacteria, respectively. Fig. S2. Symptom difference on A. thaliana seedlings treatment with B. cinerea conidia suspension. A. A. thaliana seedlings co-cultured with B. velezensis JRX-39 for 21 days and then inoculated with B. cinerea; B. control, A. thaliana seedlings cultured for 21 days on MS medium and inoculated with B. cinerea conidia suspension, showed more severe disease. Arrows indicated diseased leaves. Table S1. Identification of VOCs of the Bacillus JRX-YG39 by GC-MS. [file 12866_2022_2584_MOESM1_ESM.pdf]

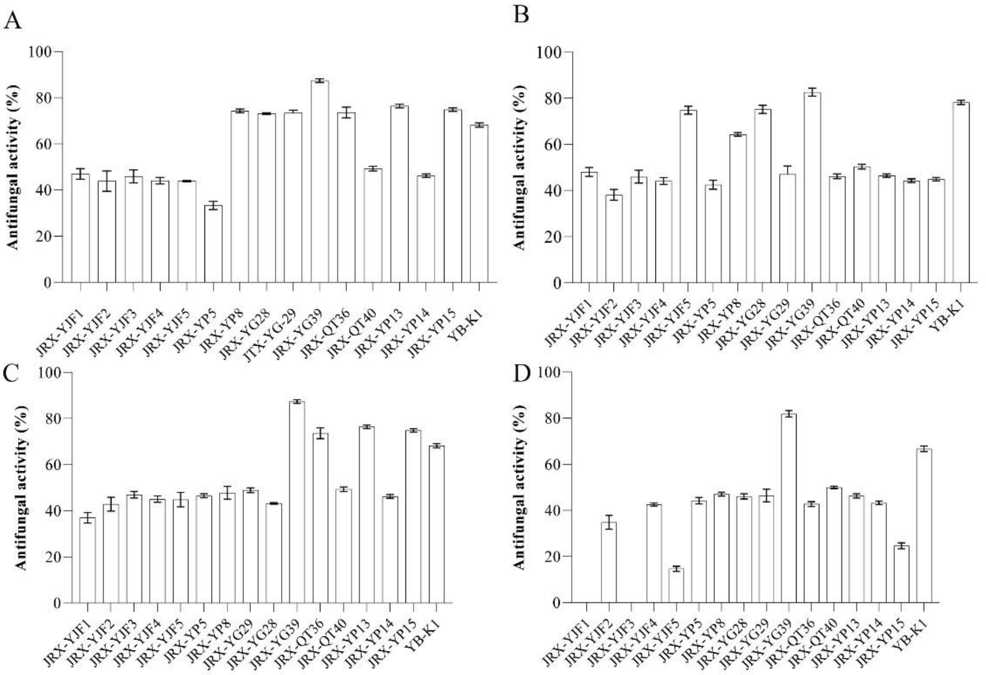

Fig.S1 Antifungal activity of 16 endophytic bacteria against four phytopathogens. A to D represented growth inhibition of *Botrytis cinerea*, *Alternaria alternata, Fusarium pernambucanum,* and *Colletotrichum gloeosporioides* after antagonist with 16 endophytic bacteria, respectively.

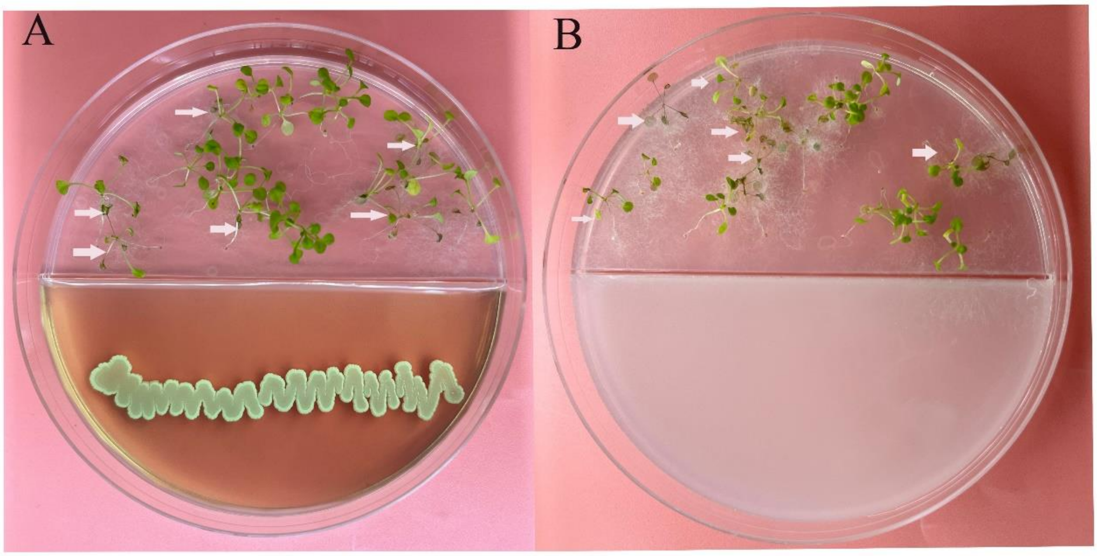

Fig. S2 Symptom difference on *A. thaliana* seedlings treatment with *B. cinerea* conidia suspension. A. *A. thaliana* seedlings co-cultured with *B. velezensis* JRX-39 for 21 days and then inoculated with *B. cinerea*; B. control, *A. thaliana* seedlings cultured for 21 days on MS medium and inoculated with *B. cinerea* conidia suspension, showed more severe disease. Arrows indicated diseased leaves.

Table S1 Identification of VOCs of the *Bacillus* JRX-YG39 by GC-MS

Compounds name	Retention time (min)	Height	Area	Area%	CAS
Acetic acid, butyl ester	2.709	189,437	3,418,832	0.164	000123-86-4
Butanoic acid, 3-methyl-	3.132	458,849	28,784,771	1.382	000503-74-2
Butanoic acid, 2-methyl-	3.278	904,510	47,121,621	2.2625	000116-53-0
phenol	4.233	792,037	14,475,054	1.295	000108-95-2
1,3-Dioxolane-4-methanol, 2,2-dimethyl-, (S)-	4.379	147,143	2,737,882	0.132	022323-82-6
Hexanoic acid	4.539	107,188	3,266,604	0.157	000142-62-1
1-Hexanol, 2-ethyl-	4.646	351,446	6,379,552	0.306	000104-76-7
1-Octanol	5.0943	143,690	3,911,473	0.188	000111-87-5
Octanoic Acid	6.229	329,926	7,544,862	0.3623	000124-07-2
Tetradecane	6.862	161,472	2,632,162	0.126	000629-59-4
2-Nonanone	7.592	130,464	3,393,606	1.163d	000821-55-6
Hexadecane	8.181	118115	6,081,545	0.292	000544-76-3
o-Acetylphenetidine	10.299	293,483	19,382,244	0.931	000581-08-8
Benzene, 1,4-dimethoxy-2,3,5,6-tetramethyl-	10.538	259,776	15,946,169	0.766	013199-54-7
1,2,5,5,6,7-Hexamethylbicyclo[4.1.0]hept-2-en-4-one	11.735	100,190	4,859,739	0.233	1000110-52-5
Hexadecanoic acid, methyl ester	12.909	170,182	10,976,802	0.527	000112-39-0
Hexadecanoic acid, ethyl ester	14.116	99,807	4,070,072	0.1954	000628-97-7
2-Cyclohexen-1-one, 4,4,5-trimethoxy-	15.708	218,632	13,995,114	0.672	056180-51-9
Hydrouracil, 1-methyl-	16.102	260,661	11,578,131	0.556	000696-11-7
L-Prolinamide	16.735	128,190	5,216,380	0.251	007531-52-4

Dibutyl phthalate	17.787	16,173,026	1,547,120,613	78.651	000084-74-2
Phenol, 3,5-dimethoxy-	18.103	1,915,821	92,293,878	4.431	000500-99-2
Dodecanamide	18.3711	207,726	12,869,982	0.618	001120-16-7
5-Chlorovaleric acid, morpholide	19.014	113,990	3,860,847	0.185	1000307-35-6
l-Leucine, N-cyclopropylcarbonyl-, hexadecyl ester	19.827	1,440,112	74,961,756	3.600	1000327-78-4
Pyrrolo[1,2-a]pyrazine-1,4-dione, hexahydro-3-(2-methylpropyl)-	19.983	1,057,526	38,915,430	1.869	005654-86-4
Bicyclo[2.2.2]octane-1-carboxylic acid	20.348	128,593	4,195,874	0.202	000699-55-8
l-Proline, N-allyloxycarbonyl-, heptadecyl ester	20.591	187142	9,242,470	0.444	1000313-66-3
Hexadecanamide	20.840	106,073	4,638,063	0.223	000629-54-9
4-Octene, 2,3,7-trimethyl-, [S-(E)]-	23.907	138,396	24,356,410	1.169	052763-13-0
9-Octadecenamide, (Z)-	24.593	530,555	34,573,353	1.66	000301-02-0
